# Microfluidic Droplet-Storage Array

**DOI:** 10.3390/mi11060608

**Published:** 2020-06-23

**Authors:** Hoon Suk Rho, Han Gardeniers

**Affiliations:** 1 Department of Instructive Biomaterials Engineering, MERLN Institute for Technology-Inspired Regenerative Medicine, Maastricht University, 6229 ER Maastricht, The Netherlands; h.rho@maastrichtuniversity.nl; 2 Mesoscale Chemical Systems Group, MESA+ Institute, University of Twente, 7522 NB Enschede, The Netherlands

**Keywords:** microfluidics, droplet array, microvalve

## Abstract

A microfluidic droplet-storage array that is capable of the continuous operation of droplet formation, storing, repositioning, retrieving, injecting and restoring is demonstrated. The microfluidic chip comprised four valve-assisted droplet generators and a 3 × 16 droplet-storage array. The integrated pneumatically actuated microvalves enable the precise control of aqueous phase dispensing, as well as carrier fluid flow path and direction for flexible manipulating water-in-oil droplets in the chip. The size of droplets formed by the valve-assisted droplet generators was validated under various operating conditions such as pressures for introducing solutions and dispensing time. In addition, flexible droplet addressing in the storage array was demonstrated by storing droplets with various numbers and compositions in different storage units as well as rearranging their stored positions. Moreover, serial injections of new droplets into a retrieved droplet from a storage unit was performed to show the potential of the platform in sequential dosing on incubated droplet-based reactors at the desired timeline. The droplet-storage array with great freedom and flexibility in droplet handling could be applied for performing complex chemical and biologic reactions, especially in which incubation and dosing steps are necessary.

## 1. Introduction

Droplet-based microfluidic systems that manipulate nano- or picoliter droplets in microchannels have been highlighted in high-throughput chemical and biologic screening with rapid and robust reactions [1,2,3,4]. With the beneficial aspects of droplet-based reactors, e.g., extremely low sample consumption as well as flexibility in the size of the reactor, enormous droplet generators have been applied for various applications, including particle synthesis [5,6,7], cell analysis and nucleic acid amplification [8,9] and sequential biochemical reactions [10,11,12]. Besides this, advanced microfabrication and microengineering techniques have been exploited to design and manufacture innovative droplet devices for combinatorial screening [13,14,15], massive droplet production [16,17,18] and accurate droplet sorting [19,20,21].

One of the fundamental aspects of the droplet-based systems for analytical chemistry and biology is preparing libraries of samples in various compositions as well as concentrations. For varying the combination and concentration of reagents in a series of droplets, microfluidic mixers were integrated with droplet generators [5,10,11]. In the combined designs, several flowing streams were emerged into a microfluidic mixer and flowed into a carrier fluid flow to form droplets with various conditions. The other concept to create concentration gradients along droplets is merging droplets by synchronizing droplets in a microchannel network [22] or using hydrodynamics with microstructures designed to trap and release droplets [13,14,23,24].

Recently, highly integrated droplet-based microfluidic arrays have been developed for combinatorial screening in a single platform [13,14,25,26,27,28,29,30]. The microfluidic platforms combined broad functional spectrum in droplet-based microfluidics, e.g., concentration gradient generation and droplet formation and storage, for building high-throughput libraries in protein crystallization [25,26], single-cell analysis [13,14,28,29] and spheroids formation [27]. In particular, static droplet arrays [24,27,28,29,30] enabled controllable droplet generation and storage, as well as multiple chemical dose to stationary droplets in a relatively simple device geometry as a promising and inexpensive alternative to conventional high-throughput screening. Although the static droplet arrays showed great potential in performing complex combinatorial screening, the reactions are based on stationary droplets with predetermined volumes. Hence, the flexible droplet size-control and droplet retrieval for further on-chip and off-chip analysis remain challenging.

After the first introduction of Quake’s valves, pneumatically actuated poly(dimethylsiloxane) (PDMS) monolithic valves fabricated by multilayer soft lithography [31], into droplet-based microfluidics [32], several multilayer PDMS devices have been presented to perform accurate handling of droplets for biochemical applications such as static fluorescence assay [33], nanoparticle synthesis [34] and single microbial cell screening [14]. The platforms showed the potential of the valve-assisted droplet generator to produce highly monodispersed droplets and combinatorial contents in a series of droplets. Furthermore, the accurate manipulation of complex fluid flows by multilayer devices, where tens- or hundreds of microvalves were integrated, in previous reports [35,36,37] showed promise to engineer an automated, multifunctional microfluidic droplet array.

Here, we discuss the development of a microfluidic droplet-storage array that enabled programmable droplet formation, multiple injections into generated droplets, addressing and incubating droplets in storage units, dosing additional droplets to stored droplets. The device comprised four valve-assisted droplet generators and an array of 48 droplet-storage units by integrating a forward–backward flow direction control and flow path changing with pneumatically actuated microfluidic valves. The flow rate ratio of a dispensing phase and a carrier fluid, as well as dispensing time, were characterized to generate water-in-oil (w/o) droplets with controlled volumes ranging from 13.2 ± 0.5 to 1204.8 ± 17.8 pL (n = 20). In addition, the repositioning of sets of stored droplets in the storage unit array was demonstrated to show the robust valve-assisted operation of flow direction and path control. Finally, the continuous operation of multiple droplet formation, storage, positioning, retrieval, and injecting was processed to present the great freedom and flexibility of the microfluidic chip in droplet handling. The microfluidic droplet-storage array may be applied for performing complex chemical and biologic reactions with tiny sample consumption, especially in which incubation and dosing steps are required, e.g., cell drug–dose response and multistep chemical synthesis.

## 2. Materials and Methods

### 2.1. Chip Design

**Figure 1:**
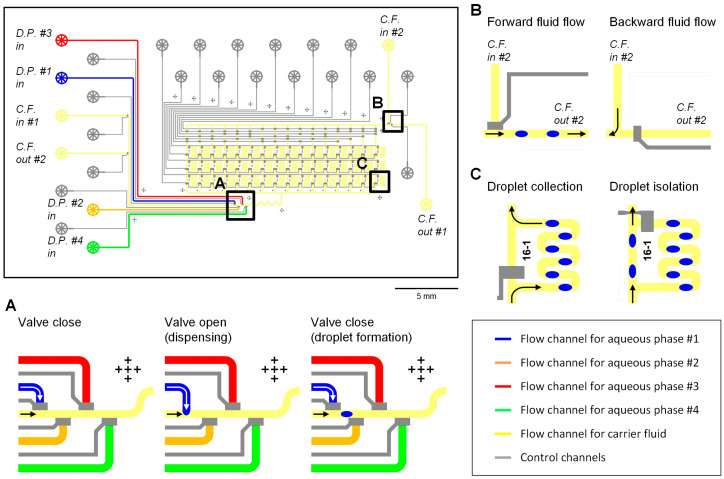
Design and operation of the microfluidic droplet-storage array. A computer-aided design shows the integration of 4 droplet generators and 48 droplet-storage units; (**A**) process flow of the droplet generation by using a microfluidic valve; (**B**) fluid flow direction control by switching a carrier fluid flow channel connection between the two sets of an inlet and an outlet; (**C**) valve operation to collect and isolate droplets in a droplet-storage unit.

The microfluidic droplet-storage array comprised 4 droplet generators and 48 droplet-storage units (Figure 1). The main carrier fluid flow channel was connected to two sets of an inlet and an outlet, and the carrier fluid flowed through the droplet generators and the storage unit array. Figure 1A shows the working process for droplet formation by using a microfluidic valve [32]. In each droplet generator, a pneumatically actuated microfluidic valve [31] was placed at the T-junction of an aqueous and the carrier fluid channels to control the connection between the two flows. The valve was normally closed to disconnect the two channels, and a volume of the aqueous phase was dispensed into the oil flow when the valve was open. Then the valve was closed again to create a segment of the aqueous phase in the carrier fluid flow. The size of the water-in-oil (w/o) droplet, the dispensed volume of the water phase, was determined by the flow rates of water and oil phases as well as the valve opening time.

Figure 1B presents the design of the inlet and outlet connection in the device for switching the flow direction of the carrier fluid (Figure 1B). On the two sides of the device, two sets of an inlet and an outlet were placed, and the channel connection was controlled by valves, which were designed at the junctions of channels. When the main oil flow channel was connected to the inlet near the droplet generators (cf. in #1) and the outlet after the incubation chambers (cf. out #1), the carrier fluid flowed from the droplet generators to incubation chambers (forward fluid flow). The oil flow direction was reversed to backward fluid flow by connecting the outlet before the droplet generators (cf. out #2) and the inlet after the chamber array (cf. in #2). The oil flow direction switching enabled the flexible addressing of droplets into the storage units and multiple droplet injections into a target droplet.

Each droplet-storage unit consisted of a bypass channel from the main carrier fluid flow channel, and two valves at the entrance and exit of the bypass channel, as illustrated in Figure 1C. By closing the valve near the bypass entrance and opening the other valve at the exit of the channel, droplets in the carrier fluid flowed into the bypass channel, the storage unit. Then, the droplets in the storage unit were trapped and isolated while the next droplets passed through the main oil channel by switching the valves on and off. The valves for isolating droplets in the 48 storage units were operated by microfluidic multiplexors [38]. The device contained 3 columns and 16 rows of the storage units within a chip dimension of 3.5 cm × 2.0 cm and 1–10 droplets, depending on the droplet size and droplet-to-droplet spacing, could be stored in each storage unit. Further integration of the storage units in a single chip would be possible by adding parallelized storage units and control channels for high-throughput analysis.

### 2.2. Chip Fabrication

The microfluidic device was fabricated by multilayer soft lithography technique [31,39], and we followed a modified fabrication protocol based on our previous studies [36,37]. The PDMS device consisted of a top fluidic layer and a bottom control layer; the heights of fluid flow channels and control channels were 38 ± 2 μm and 18 ± 2 μm (n = 10), respectively.

### 2.3. Chip Operation

A pneumatic control system was used for operating fluid flow in the microfluidic droplet-storage array. Reagents were loaded into the flow channels by applying pressure from the backside of solutions [40], and microvalves were actuated by applying compressed nitrogen gas into the control ports. The pneumatic control system was automated by interconnecting 3/2-way solenoid valve manifolds, precision pressure regulators and an EasyPort USB digital I/O controller (all from Festo, Delft, The Netherlands) and controlled by a LabVIEW program (National Instruments Co., Austin, TX, USA). The valve operation for changing the fluid flow direction and droplet injection was controlled by sequencing pre-measured droplet moving time. However, a feedback control strategy may enable real-time sequencing by integrating a digital video processing software [41] with the operating setup.

### 2.4. Materials

Food dye solutions filtered with a 0.2-µm syringe filter (Whatman PLC, Sigma-Aldrich, Zwijndrecht, The Netherlands) and mineral oil containing 1.5% (*w/w*) Span 80 (all from Sigma-Aldrich, Zwijndrecht, The Netherlands) were used as the aqueous phases and the carrier fluid, respectively, for the generation of water-in-oil droplets. For sequential dilution by serial droplet injection, 1-g/L rhodamine B isothiocyanate-dextran (RITC-dextran, average molecule weight ~10,000, Sigma-Aldrich Chemie BV, Zwijndrecht, Netherlands) prepared in Milli-Q water (Millipore Co.) and Milli-Q water were used as a stock solution and a diluent, respectively.

### 2.5. Data Processing

A stereomicroscope (Motic SMZ171-TLED, LabAgency Benelux B.V., Dordrecht, The Netherlands) equipped with a CMOS camera (Moticam 3.0) and an inverted fluorescence microscope (Olympus IX73, Olympus Netherlands BV, Leiderdorp, The Netherlands) installed with a digital camera (ORCA-ER, Hamamatsu Photonics Deutschland GmbH, Herrsching, Germany) and an automatic XY-stage (99S000, Ludl Electronic Products, Ltd., NY, USA), were used for monitoring droplet generation and addressing in the device. An N 2.1 filter cube (excitation: BP 515–560 nm; emission: LP 590 nm) was used for observing RITC-dextran fluorescence signals with the fluorescence microscope. Acquired images and recorded movies were processed and analyzed by VirtualDub software (http://www.virtualdub.org/) and Image J software (http://rsb.info.nih.gov/ij/).

## 3. Results and Discussion

### 3.1. Droplet Generation with a Pneumatically Actuated Valve

**Figure 2:**
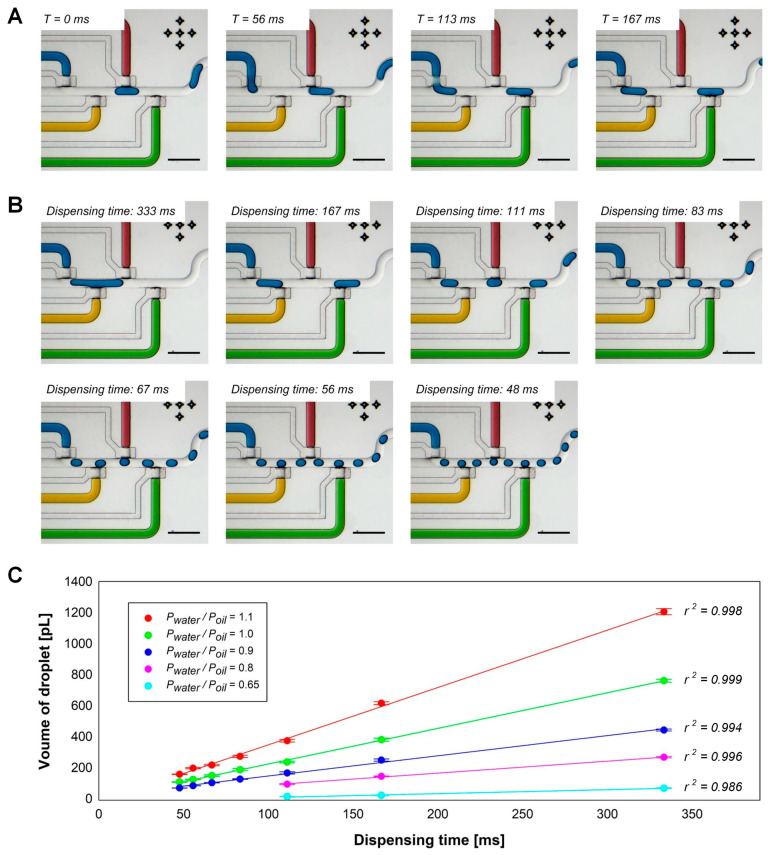
The w/o droplet generation with a pneumatically actuated microfluidic valve; (**A**) Step-by-step procedures of droplet formation at the constant droplet generation condition (P_water_/P_oil_ = 1 and dispensing time = 167 ms); (**B**) Droplet size-control by varying the dispensing time at the constant fluid flow condition (Pwater/Poil = 1). Images captured from a recorded movie. Scale bars: 400 µm; (**C**) relationship between the dispensing time and the volume of formed droplets at various fluid flow conditions (n = 20).

The w/o droplets were formed at the T-junction of the aqueous phase and carrier fluid channels in the device by using a pneumatically actuated valve [32]. The sequence of the valve-assisted droplet formation in the droplet generator is shown in the time–series images in Figure 2A. Initially, pressures were applied for loading the aqueous phase and carrier fluid in the channels; however, the water flow was seized by closing the valve in the entrance of the aqueous phase channel. When the valve was open, the aqueous phase flowed into the main carrier fluid channel until the valve is closed again. Consequently, the dispensed volume of the aqueous phase created a water droplet in the oil flow. Hence, the size of the droplet was determined by the applied pressure for water and oil flows, as well as the opening time of the valve. The applied pressure ratio for water and oil flows (Pwater/Poil) and dispensing time for the droplet generation in Figure 2A are 1 and 167 ms, respectively.

For the calibration of operating conditions of the droplet generator, we created droplets under various dispensing times at constant applied pressure for water and oil phases, and different applied pressure ratios of water flow to oil flow while dispensing time was kept constant. Figure 2B shows droplet formation with various dispensing times ranging from 48 ms to 333 ms at a constant fluid flow condition (P_water_/P_oil_ = 1). As an increased dispensing time, the formed droplet-size linearly increased. The relationship between the dispensing time and droplet volume at various fluid flow conditions is shown in Figure 2C (n = 20). The linear regressions with relatively small standard deviations represent the accurate droplet size-control and monodispersity of the formed droplets in the valve-assisted droplet generator. Supplementary Video S1 in Supplementary Materials shows the demonstration of valve-assisted droplet formation by varying dispensing time continuously.

### 3.2. Droplet Addressing by the Fluid Flow Direction Control

**Figure 3:**
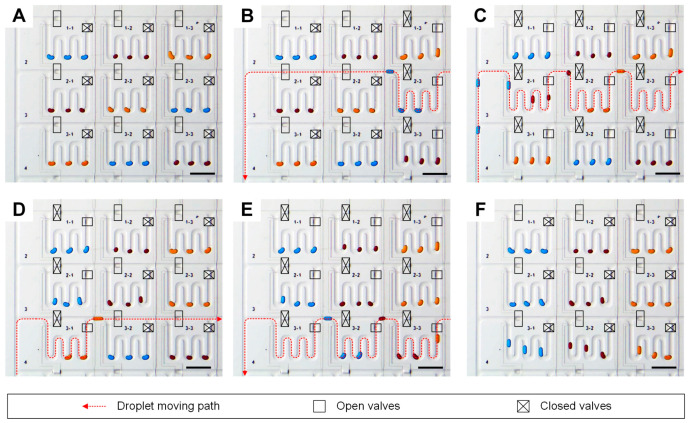
Droplet addressing in the storage unit array. (**A**) Nine sets of three droplets were isolated in a 3 × 3 storage unit array with random orders in droplet colors. (**B**–**E**) By moving the droplets with the control of carrier fluid flow direction as well as droplet moving path, (**F**) the color orders of droplets in each row rearranged to blue, red and yellow. Scale bars: 500 µm.

For demonstrating the droplet addressing capability of our microfluidic device, we relocated collected droplets in droplet-storage units (Figure 3). We generated and isolated nine sets of three water droplets formed with three different colored dye solutions, blue, red and yellow, into nine storage units. Initially, storage units in the first row, 1–1, 1–2 and 1–3, the second row, 2–1, 2–2 and 2–3, and the third rows, 3–1, 3–2 and 3–3, were filled with blue, red, yellow droplets, red, yellow and blue droplets and yellow, blue and red droplets, respectively (Figure 3A). Each storage unit contained one set of valves for bypass and collection of droplets into the unit, and the moving path of droplets was determined by the valve actuations. The flow direction of the carrier fluid was controlled by switching the connection of the two sets of an inlet and an outlet. For example, the droplets were moved to forward direction when the inlet of the carrier fluid near droplet generators and the outlet of the oil phase behind the chamber array were connected. On the contrast, the carrier fluid flowed backward with the connection of the oil inlet behind the chambers and the oil outlet closed to the droplet generators. For relocating droplets in storage units in the second row, blue droplets were retrieved from the storage unit 2–3 by connecting the bypass lines of 2–1 and 2–2 and the collection line of 2–3 with backward carrier fluid flow (Figure 3B). Then, the carrier fluid flow direction was changed to forward while the collection lines of 2–1, 2–2 and 2–3 were connected for collecting blue, red and yellow droplets in storage units 2–1, 2–2 and 2–3 (Figure 3C). By repeating the processes the yellow droplets in storage unit 3–1 were retrieved (Figure 3D) and pushed blue and red droplets in the storages 3–2 and 3–3 (Figure 3E) to make the same color order of stocked droplets, blue, red and yellow through the first, second and third columns (Figure 3F). The demonstration of relocating droplets in the storage units shows the capability of the device in collecting droplets in the array as well as freedom in setting the order of droplet-based reactors. The Supplementary Video S2 shows the procedure in real time.

### 3.3. Serial Injection of Droplets into a Target Droplet

In the most chemical and biologic experiments, dilution and mixing of samples are fundamental operations for preparing, processing and analyzing reactions. Merging droplets is one of the most useful and practical operations in droplet-based reactors to vary the compositions and concentration of reagents for performing complex reactions [13,14,22,23,24,34]. Droplet-based microfluidic devices with continuous flows controlled the droplet merging by sequencing droplet formations [22] or integrating microstructures where droplets were trapped by hydrodynamics [13,14,23,24]. In valve-assisted droplet-based microfluidics, droplet merging was operated by synchronizing valve operation of in-line droplet generators [34].

**Figure 4:**
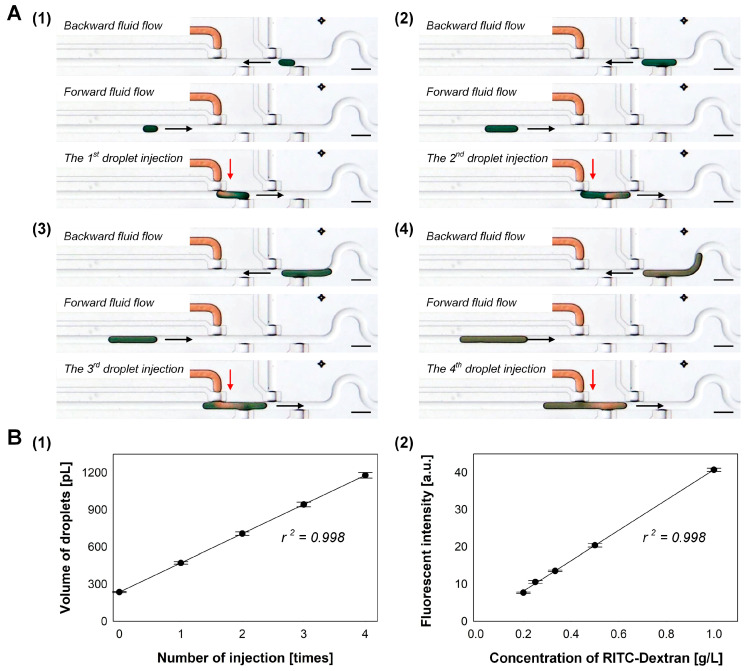
Serial injection of droplets into the target droplet in the droplet generator; (**A**) Process flow of the serial injection by changing the flow direction of the carrier fluid. Scale bars: 500 µm; (**B**) sequential dilution of RITC-dextran droplet (initial concentration of 1 g/L) by adding multiple Milli-Q water droplets (n = 3).

The droplet-injection in our microfluidic droplet-storage array device is based on the synchronization of droplet generators; however, the forward–backward flow direction control on the carrier fluid enabled multiple injections of droplets into a formed droplet in a single droplet generator. Figure 4A and Video S3 in Supplementary Materials show the operating procedure to perform serial droplet-injection into a target droplet. After the formation of a blue droplet, the droplet flowed back to the droplet generators by changing the flow direction of the carrier fluid to backward. Then, the oil flow direction was switched to forward direction again, and a red droplet was dispensed into the blue droplet. The same process was repeated for the sequential injection of red droplets into the blue droplet. Figure 4B shows the serial dilution of RITC-dextran by injecting Mill-Q water droplets into a preformed droplet containing 1-g/L RITC-dextran. The applied pressure ratio of water flow to oil flow and dispensing time were 1 and 111 ms, respectively. By repeating the Milli-Q droplet injection, the volume of the RITC-dextran droplet linearly increased with an increase of 236 ± 4 pL (Figure 4B(1)). Figure 4B(2) shows the relationship between the calculated concentration and measured the fluorescence intensity of the RITC-dextran droplet in the sequential dilution. The RITC-dextran fluorescence intensity in the droplet linearly decreased as droplet volume increased by the serial injection of Milli-Q water droplets.

### 3.4. Continuous Processing of Droplet Formation, Addressing and Injection

To demonstrate the feasibility of the microfluidic device for creating a desired library of droplets in the storage unit array, we continuously processed multiple droplet generations with different numbers and reagents and droplet addressing in different storage units. In addition, we performed repositioning of droplets as well as injecting a new droplet into the droplet retrieved from the storage unit by controlling carrier fluid flow directions with valve operation.

**Figure 5:**
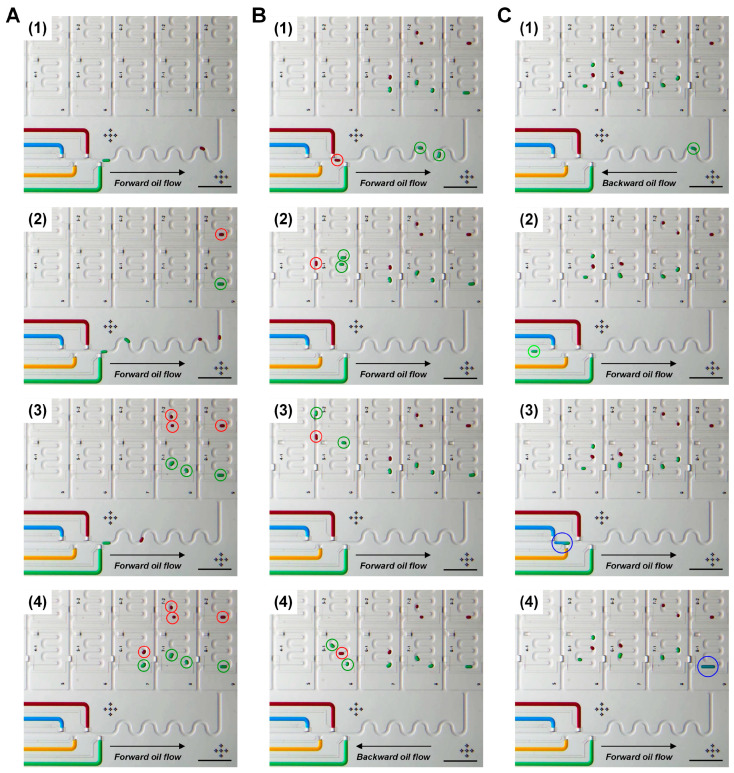
Continuous processes of (**A**) droplet formation and storing, (**B**) repositioning and (**C**) injection and restoring. Scale bars: 1 mm.

Flexible droplet generation and storing in the microfluidic droplet-storage array is shown in Figure 5A. First, one red droplet and one green droplet were formed and collected in the storage units, 8–2 and 8–1 (Figure 5A(1,2)), then two red and two green droplets were generated and positioned in the storage units in the next row, 7–2 and 7–1 (Figure 5A(3)). The following droplets, one red and one green, were placed in the storage unit 6–1 together (Figure 5A(4)). Figure 5B shows the rearranging of the order of droplets by switching the oil flow path and flow. After generating two green droplets, a red droplet was formed and followed the green droplets. The green droplets were trapped in the storage unit 5–1, while the red droplet was flowed in the main oil channel next to the storage. Then we flowed one green droplet and inserted the red droplets between the two green droplets. Finally, the three droplets were isolated in the storage unit 5–1, in a new order, green–red–green. To demonstrate dosing a reagent into an incubated droplet, we added a blue droplet into the stored green droplet (Figure 5C). The forward–backward oil flow direction control enabled the operations of retrieving the green droplet from the storage unit (Figure 5C(1)), placing to droplet generator (Figure 5C(2)), adding a new droplet (Figure 5C(3)) and restoring into the storage unit (Figure 5C(4)). The Video S4 in Supplementary Materials shows the real-time operation of the processes.

The other attractive function of the device for long-term incubation of droplets in the storage array is shaking droplets. The pristine PDMS shows a hydrophobic characteristic with a water contact angle of 100–112° [42]; however, the wetting of the aqueous phase onto the PDMS surface is still challenging for PDMS-based w/o droplet generators [43]. Although PDMS surface treatment with a commercial water repellent Aquapel [43,44] may reduce the water wetting on the PDMS surface, this non-permanent treatment is limited for the long-term storage of water droplets in PDMS channel without the loss of the water droplet volume. Our microfluidic droplet-storage device is capable of switching the main oil flow direction continuously by automated valve operation. Hence, water droplets collected in storage units can be shaken to prevent droplet settling in contact with the PDMS surface that may result in reducing the droplet volume during long-term incubation (Video S5 in Supplementary Materials). In non-treated PDMS channels, the volume loss of w/o droplets was 25% ± 3% of the initial volume of droplets with shaking while the droplet volume reduced 73% ± 2% without shaking after 5 h incubation at room temperature (Figure S1 in Supplementary Materials). Furthermore, the function of droplet shaking may be useful for conducting biochemical reactions, where agitation plays a critical role, such as protein fibrillation and aggregation [12,45,46].

## 4. Conclusions

In this work, we established a microfluidic droplet incubation chamber array to combine programmable droplet formation, multiple injections into formed droplets, addressing droplets into incubation chambers, dosing additional droplets to incubated droplets in a single device. All the droplet handling processes were performed by automated microfluidic valve control. Droplet formation with an accurate size control was validated under various valve operating conditions and applied pressure for loading solutions. In addition, flexible droplet addressing and multiple dosing on a formed droplet were demonstrated by multiplexing integrated microvalves and the forward–backward flow direction control. The microfluidic droplet incubation chamber array may be an attractive tool for performing complex chemical and biologic reactions with extremely small sample consumption, particularly in which incubation and dosing steps are required.

## Supplementary Materials

The following are available online at https://www.mdpi.com/2072-666X/11/6/608/s1, Video S1: Valve-assisted droplet formation, Video S2: Droplet addressing in a storage array, Video S3: Serial injection of droplets, Video S4: Journey of droplets on a chip, Video S5: Shaking droplets in a storage unit and Figure S1: Volume loss of w/o droplets in a non-treated PDMS channel.
