# Supplementary material for: Microfluidic Droplet-Storage Array
Source: Micromachines (Basel). 2020 Jun 23;11(6):608. doi: 10.3390/mi11060608 (PMC7344826; doi:10.3390/mi11060608)
Supplement: Supplementary file 1 [file micromachines-11-00608-s001.zip › micromachines-836187 supplementary materials/FigureS1.pdf]

Supplementary Materials

Microfluidic Droplet-Storage Array

Hoon Suk Rho [1,2] and Han Gardeniers [2,*]

1 Department of Instructive Biomaterials Engineering, MERLN Institute for Technology-Inspired Regenerative Medicine, Maastricht University, 6229 ER Maastricht, The Netherlands; h.rho@maastrichtuniversity.nl
2 Mesoscale Chemical Systems Group, MESA+ Institute, University of Twente, 7522 NB Enschede, The Netherlands
* Correspondence: j.g.e.gardeniers@utwente.nl; Tel.: +31-(0)53-489-4356

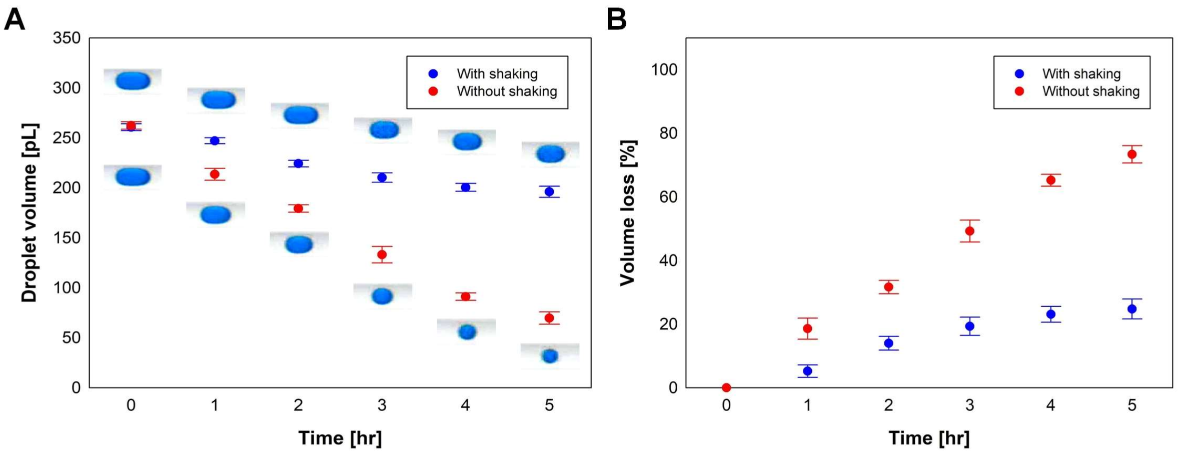

Figure 1. Volume loss of w/o droplets in a non-treated PDMS channel. Three w/o droplets were generated and stored in a storage unit in the microfluidic droplet storage array. Then, the volume change (**A**) and volume loss rate (**B**) of the droplets were measured during 5 h incubation at room temperature with shaking (blue) and without shaking (red) (n = 3).
